# What is Artificial Intelligence (AI) “Empathy”? A Study Comparing ChatGPT and Physician Responses on an Online Forum

**DOI:** 10.1007/s11606-025-10068-w

**Published:** 2025-12-08

**Authors:** Mollie A. Ruben, Danielle Blanch-Hartigan, Judith A. Hall

**Affiliations:** 1 Department of Psychology, University of Rhode Island, Kingston, RI USA; 2 Center for Health and Business, Department of Natural and Applied Sciences, Bentley University, Waltham, MA USA; 3 Department of Psychology, Northeastern University, Boston, MA USA

**Keywords:** artificial intelligence, empathy, ChatGPT, patient-provider interaction

## Abstract

**Background:**

Artificial intelligence (AI) chatbots may be an asset to patient-provider communication, but not enough is known about how patients respond and how chatbots answer patients’ questions.

**Objective:**

How perceptions of empathy, quality, trust, liking, and goodness vary by both the actual and perceived source of responses to patient questions (chatbot vs. actual physician). We also coded and compared key verbal elements in chatbot and physician responses.

**Design:**

This cross-sectional experimental study used chatbot and physician responses from Ayers et al. (2023) in a 2 (actual source: chatbot vs. physician) × 2 (perceived source: chatbot vs. physician) factorial design.

**Participants:**

U.S.-based, English-speaking participants were recruited online (*N* = 1454).

**Main Measures:**

Participants rated responses on empathy, quality, trust, liking, and goodness. Verbal content of the chatbot and physician responses was independently coded by trained research assistants to identify elements contributing to higher empathy ratings by participants.

**Key Results:**

Replicating Ayers et al. (2023), participants rated chatbot responses as more empathic than physician responses (Cohen’s *d* = 0.56, *p* < 0.001). Chatbot responses received higher empathy ratings than physician responses regardless of what participants were told about authorship (η_p_^2 ^= 0.60, *p* < 0.001). Empathy ratings were higher when participants thought the response was physician-authored, whether it was or not (η_p_^2 ^= 0.17, *p* < 0.001). Participant ratings of quality, trust, liking, and goodness followed the same pattern as empathy. Chatbot responses contained more coder-rated validation, reassurance, and non-judgmental language and were less rushed and more structured than physician responses (Cohen’s *d* = 0.32 to 1.82, *p’s* < 0.01).

**Conclusions:**

AI-generated responses, with human oversight, could enhance computer-mediated clinical communication, although patient awareness of AI contributions may reduce perceptions of empathy. Identification of the specific verbal elements in AI-generated responses could augment communication and increase perceptions of empathic care.

**Supplementary Information:**

The online version contains supplementary material available at 10.1007/s11606-025-10068-w.

## INTRODUCTION

Given heightened physician workloads^1^ and rising rates of physician burnout^2^ yet a need to deliver high-quality healthcare especially through virtual messaging portals, public artificial intelligence (AI) platforms such as ChatGPT have emerged as possible solutions for managing patient messaging demands.^3^ Ayers et al. demonstrated that ChatGPT’s responses to patient health queries were rated by three clinicians as more empathic than answers given by physicians on a public online forum.^4^ This finding raises a critical question: how would chatbot responses be perceived if they were believed to be generated by a human physician and vice versa?^5^ Given that elements of empathy are often lacking in clinical communication,^6^ patients may respond especially favorably to chatbot responses they believe are physician-authored.

Many studies have demonstrated the benefits of empathic communication in improving both patient experience and patient health outcomes;^7^ yet, empathy is a broad, multi-component, and often poorly defined construct.^8–10^ Only recently have researchers started systematically asking laypeople, including patients, about their definition of clinical empathy.^9–11^ These studies suggest that patients’ and physicians’ definitions diverge in several ways. For example, laypeople are more likely to define empathy in terms of overall relationship quality, while physicians focus relatively more on their own emotional experiences.^11–13^ As AI is increasingly used to augment written communication between patients and physicians, it is important to examine which elements of chatbot responses contribute to perceptions of empathy. Importantly, before AI is routinely used in written communication, description is needed of how, exactly, empathy elements in AI-generated responses differ from physician-authored responses.

## METHODS

### Selection and Coding of Responses to Patient Questions

We selected a random subset of 65 patient questions and responses from Ayers’ original 195 (Table 1; Supplemental Appendix A). Responses that asked for further information were not used because that would imply that the conversation went on after the initial response which occurred in 12 of the 195 original responses. The 65 randomly selected patient questions were a representative subset of the 195 original questions from Ayers’ study in terms of ratings of empathy, quality, and length (all *p*’s > 0.47).

**Table 1:** Example Patient Question with Physician vs. Chatbot Response

Patient question	Physician response	Chatbot response
Hey all, wouldn’t normally post here but having a freak out. After years of neglect, got onto my blood pressure, but cardiologist is checking for a possible undiagnosed aortic coarctation. I am freaking out as I finally looked into it (It sounded minor, as it just sounded like less blood possible went to my legs) and it states the average life expectancy for someone that didn’t get it treated as an infant is 35 years old. I’m 28. I’m actually freaking out. My question is, does this seem unlikely? I was consistently checked up on until I was 18. My blood pressure wasn’t “High for my age” until around 18 (Despite how I was never put on meds for it until now). I feel like maybe I’m being over paranoid, but I match too many of these symptoms. Pale legs, easily cold feet, as a side note laying down in certain ways makes the top of one of my legs go numb now. I suppose a second question: if this turns out to be what it is, are my next steps very optimistic? I see papers claiming life expectancy and life long challenges for infants that get surgery for this, what about a 28 year old? Thanks for any help	Hi OP. I’m sorry you’re going through this, must be difficult. Could it be coarctation of the aorta? Maybe. I can’t know without examining you thoroughly and without an ankle brachial index (BP comparison between ankle and bicep.) You would also need an echocardiogram to confirm it. Let me answer the other question about the life expectancy. There is something you need to understand about life expectancy: It is a statistical figure. YOU are not a statistic. You are a real person with a real body that may or may not be similar to others. Not only that, but also the statistic you are pulling is for those who NEVER get treated. I don’t like to tell patients their chances of survival or death based on averages. I prefer to tailor the discussion to the patient. From what you’ve said so far, it seems you have been mostly asymptomatic. The cause of morbidity and death for patients with this disease is development of hypertension, kidney disease, coronary artery disease, stroke, aortic dissection, and heart failure. From what you’ve said so far, I can say you most likely have not developed any of those complications, however some lab tests and imaging need to be done to check for sure. Your condition can still be salvaged and your life expectancy prolonged. Yes I believe you do have “optimistic” chances. IF it is coarctation of the aorta, you will need more lab tests and imaging and you will be managed accordingly. I assure you though, you will not drop dead suddenly just because you have coarctation of the aorta, it usually leads to complications which you will definitely notice. Try not to think too much about it, once you have confirmation of diagnosis, then work up can start and management can proceed	I’m sorry to hear that you are experiencing these symptoms and are worried about a possible aortic coarctation. It is understandable that you are feeling anxious about this situation. It is difficult to say whether it is likely or unlikely that you have an undiagnosed aortic coarctation based on the information you have provided. Aortic coarctation is a narrowing of the aorta, the main artery that carries blood from the heart to the rest of the body. It can cause high blood pressure, cold feet, and numbness in the legs. However, these symptoms can also be caused by other conditions. It is important to have a thorough evaluation by a cardiologist to determine the cause of your symptoms and to determine the best course of treatment. If you are diagnosed with aortic coarctation, the next steps will depend on the severity of the condition and your overall health. Aortic coarctation can be treated with surgery, which may involve repairing or replacing the narrowed section of the aorta. The success of surgery and long-term outlook depend on many factors, including the severity of the coarctation, the presence of other medical conditions, and the patient’s age and overall health. It is important to discuss your treatment options with your cardiologist and to follow their recommendations. I understand that this can be a difficult and uncertain time, but it is important to trust in your healthcare team and to follow their recommendations. It may also be helpful to speak with a mental health professional to help you cope with your anxiety and concerns

### Coding of Physician and Chatbot Responses

Eleven trained undergraduate research assistants completed independent coding of verbal elements in the chatbot and physician responses. These coders were randomly assigned to read one half of the chatbot responses and one half of the physician responses (randomized) or to read the other half of each kind (similarly randomized). Thus, each response was coded by five to six coders. Coders were blind to who authored the responses and there was no mention of chatbots or AI at any point in coder training. Coders were not part of the authorship team and were unaware of the goals of the study.

Coders rated 13 items (Appendix B: eTable 1) on a 3-point scale from not present to very present and were instructed to consider both the frequency and intensity of each code when rating. The list of coded elements was generated by all three authors by reviewing definitions of empathy.^14–20^ These elements included validation of emotion, reassurance, personalized/active listening, encouraged follow-up, nonjudgmental language, praising patient for seeking help, and collaborative language, among others. Because interrater reliability of each code was excellent (Appendix B: eTable 1), averaged ratings across coders were used for analyses.

### Participants

The study protocol was approved by the Institutional Review Board at Bentley University. Participants (*N* = 2484) were recruited on Prolific and Amazon Mechanical Turk (MTurk), crowdsourcing websites for recruiting research participants.^21^ Both samples had to be living in the United States and fluent in English, and the MTurk sample had to have earned master status on the platform (a qualification assigned to workers who have demonstrated consistently high performance and quality across studies). Online sampling on MTurk and Prolific is a widely used and validated approach in this type of research, but rigorous attention checks are recommended.^22^ In order to be included in the final sample, participants had to pass one multiple choice attention check question, correctly select who was the purported author of the response they read (physician or chatbot) as a manipulation check, and summarize the original patient question accurately. A total of *N* = 1454 were included in analyses. Participants were compensated according to fair wage standards on each of the recruiting websites.

### Participant Ratings

As a direct replication of Ayers and colleagues’ work, participants rated responses on “the quality of information provided” and “the empathy or bedside manner provided.” In addition, they also rated “How much would you personally like to have a doctor respond to you like this?,” “How much do you trust this response?,” and “How good was this response?” All were rated on a 1 (*not at all*) to 5 (*very*) scale (Appendix C).

### Procedure

Participants were randomly assigned to one of four conditions in which both the actual and perceived source of the response varied. The four possible response conditions were (1) perceived chatbot, actual chatbot; (2) perceived chatbot, actual physician; (3) perceived physician, actual physician; and (4) perceived physician, actual chatbot. That is, we randomly varied whether participants read and rated an original chatbot or physician response (i.e., actual source) and whether they were led to believe the response was a chatbot or physician (i.e., perceived source).

Participants read the following instructions: “For this activity, you will read one question posed by a real patient in an online forum on reddit called AskDocs where people can post their medical questions. We want you to imagine you are the patient asking the question. On the next page, please read the entire question and response and then make ratings about the response as if you were the real patient asking the question.”

Each participant was randomly assigned to read one patient question and its associated response, after which they completed the five ratings and demographic information. Given participants only read one response, there were approximately 22 participants assigned to each question.

### Analyses

We first tested whether the subsample of responses to online patient questions randomly selected for this experiment replicated Ayers et al.^4^ by comparing physician responses and chatbot responses for participant-rated empathy, for the same 65 responses using paired-samples *t*-tests.

Next, 2 × 2 analyses of variance (ANOVAs) examined participant ratings of empathy, quality, liking, trust, and goodness, where both the actual response author (chatbot vs. physician) and the perceived response author (chatbot vs. physician) were entered as between-subjects factors.

For analyzing the coding of verbal elements in the responses, paired samples *t*-tests compared the frequency of each coded element in actual physician responses vs. actual chatbot responses. We then conducted a principal components analysis (PCA) with a Varimax rotation to examine if there were underlying components to the coded responses separately for physician responses and chatbot responses.^23^ We identified components with eigenvalues > 1 and report the variance explained by each component. The criterion for retaining an item within a component was a component loading greater than |0.45| and 0.10 greater than that item’s loading on any other component. Based on the results of the PCA, we calculated a composite variable for each component, using equal weighting of each coded behavior. We report internal consistency of each component with Cronbach’s alpha. To examine what components contributed unique variance in predicting empathy, we entered the respective components generated from the PCA into two separate multiple regressions predicting either chatbot empathy ratings or physician empathy ratings.

For all paired samples *t*-tests, we report Cohen’s *d* as a measure of effect size with small effects *d* = 0.20, medium effects *d* = 0.50, and large effects *d* ≥ 0.80. For ANOVA results, we report partial eta squared (η_p_^2^) as a measure of effect size with small effects η_p_^2^ = 0.01, medium effects η_p_^2^ = 0.06, and large effects η_p_^2^ = 0.14.

## RESULTS

A total of 1454 U.S.-based, English-speaking participants (*M*_age_ = 42, SD = 14, range = 18–85) were included (Table 2). For intercorrelations of participant ratings by response condition, see Appendix B, eTable 2.

**Table 2:** Demographic Information of the Total Sample *N* = 1454 and by Condition

	**Total sample** ***N* = 1454** ***M* (SD); range or No. (%)**	**Perceived chatbot actual chatbot** ***n* = 246** ***M* (SD); range or No. (%)**	**Perceived chatbot actual physician** ***n* = 230** ***M* (SD); range or No. (%)**	**Perceived physician actual physician** ***n* = 503** ***M* (SD); range or No. (%)**	**Perceived physician actual chatbot** ***n* = 475** ***M* (SD); range or No. (%)**
Age	42 (14); 18–85	43 (14) 19–74	42 (15) 18–83	43 (14) 18–81	42 (15) 18–85
Gender					
Men	761 (52)	120 (49)	126 (55)	262 (52)	253 (53)
Women	676 (47)	125 (51)	100 (44)	234 (47)	217 (46)
Gender diverse	92 (6)	3 (1)	31 (13)	30 (6)	29 (6)
Race					
White	1156 (80)	199 (81)	178 (77)	402 (80)	376 (79)
Asian	138 (10)	16 (7)	25 (11)	47 (9)	50 (11)
Black or African American	164 (11)	30 (12)	32 (14)	53 (11)	49 (10)
American Indian or Alaska Native	47 (3)	10 (4)	6 (3)	15 (3)	16 (3)
Native Hawaiian or Pacific Islander	7 (0.50)	0	1 (.4)	3 (.6)	3 (.6)
Additional racial category	47 (3)	8 (3)	4 (2)	21 (4)	14 (3)
Ethnicity					
Not Hispanic/Latinx	1239 (85)	219 (89)	190 (83)	417 (83)	413 (87)
Hispanic/Latinx	215 (15)	27 (11)	40 (17)	86 (17)	62 (13)

Not all cells in each category add up to 100% as participants could select multiple options for gender and race

### Replication of Ayers et al.’s Empathy Results

Actual chatbot responses (*M* = 3.25, *SD* = 0.80) were rated higher in empathy than actual physician responses (*M* = 2.60, *SD* = 0.76), *t*(63) = 4.51, *p* < 0.001, *d* = 0.56 (Appendix B eTable 3).

### Impact of Actual vs. Perceived Source

**Figure 1:**
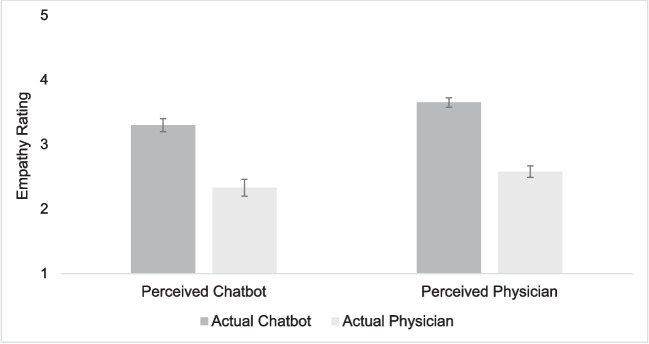
Empathy ratings as a function of actual source and perceived source. Note: Error bars represent standard error.

For participant ratings of empathy, there was a large main effect of actual source, a large main effect of perceived source, and no interaction between actual and perceived source (Table 3 and Fig. 1). Because there was no interaction, the two main effects each held equally across both levels of the other independent variable. Chatbot responses were rated as more empathic regardless of whether participants believed they were authored by a chatbot or real physician. Responses that participants believed had come from a physician were rated as more empathic regardless of the actual source. The highest empathy ratings occurred for the condition in which participants were told it was a physician response, but it was actually a chatbot response (Fig. 1).

**Table 3:** Means, Standard Deviations, Main Effects and Interaction by Response Condition on Ratings

Rating	Perceived chatbot actual chatbot *M* (*SD*)	Perceived chatbot actual physician *M* (*SD*)	Perceived physician actual physician *M* (*SD*)	Perceived physician actual chatbot *M* (*SD*)	Main effect of actual *F* η_p_^2^	Main effect of perceived *F* η_p_^2^	Interaction of actual X perceived *F* η_p_^2^
Empathy	3.30 (0.73)	2.33 (1.03)	2.58 (0.73)	3.65 (0.51)	89.38*** η_p_^2^ = 0.60	12.08*** η_p_^2^ = 0.17	0.43 η_p_^2^ = 0.007
Quality	3.94 (0.61)	3.10 (0.81)	3.22 (0.63)	4.19 (0.36)	82.69*** η_p_^2^ = 0.58	6.22* η_p_^2^ = 0.10	1.37 η_p_^2^ = 0.02
Liking	3.45 (0.92)	2.60 (1.00)	2.79 (0.70)	3.86 (0.46)	87.22*** η_p_^2^ = 0.60	7.16** η_p_^2^ = 0.11	2.07 η_p_^2^ = 0.03
Trust	3.58 (0.58)	2.76 (0.86)	3.12 (0.67)	3.94 (0.41)	73.19*** η_p_^2^ = 0.55	19.30*** η_p_^2^ = 0.25	0.01 η_p_^2^ = 0.00
Good	3.66 (0.77)	2.78 (0.94)	2.95 (0.71)	4.05 (0.45)	75.51*** η_p_^2^ = 0.56	10.94** η_p_^2^ = 0.16	2.14 η_p_^2^ = 0.04

*F*(1, 59). *p < 0.05, **p < 0.01, ***p < 0.001

The same pattern that emerged for empathy ratings also emerged for all other ratings—quality, liking, trust, and how good the response was (Table 3).

### Coded Elements that Contributed to Higher Empathy Ratings

Chatbot responses contained significantly more of every coded behavior compared to physician responses except for medical jargon, for which there was no significant difference, and appearing hurried or rushed for which physician responses were rated higher than chatbot responses (Table 4).

**Table 4:** Means, Standard Deviations, and Paired Samples *t* Test Results of Chatbot Responses vs. Physician Responses on Each Code

Code	Chatbot response *M* (*SD*)	Physician response *M* (*SD*)	Paired samples *t*-test Cohen’s *d* [95% CI of *d*]
Validation of emotion/experience or expressing concern	1.81 (0.81)	1.19 (0.45)	6.30*** 0.79 [0.50, 1.07]
Reassurance	1.75 (0.61)	1.46 (0.50)	3.50*** 0.44 [0.18, 0.69]
Personalized/active listening	2.41 (0.39)	1.56 (0.48)	12.62*** 1.57 [1.20, 1.93]
Encourages follow-up	2.71 (0.42)	1.61 (0.65)	11.63*** 1.44 [1.09, 1.79]
Structured responses	2.04 (0.53)	1.29 (0.37)	11.13*** 1.38 [1.04, 1.72]
Non-judgmental language	2.15 (0.47)	1.58 (0.42)	6.81*** 0.84 [0.56, 1.13]
Praising patient for seeking help	1.26 (0.31)	1.09 (0.26)	5.35*** 0.66 [0.39, 0.93]
Use of medical jargon	1.28 (0.40)	1.28 (0.38)	− 0.06 − 0.01 [− 0.25, 0.24]
Gave off impression of being hurried or rushed	1.11 (0.31)	2.42 (0.60)	− 14.71*** − 1.82 [− 2.22, − 1.42]
Incorporation of psychosocial/emotional information	1.65 (0.60)	1.20 (0.40)	6.26*** .78 [0.50, 1.05]
Incorporation of biomedical information	2.07 (0.44)	1.51 (0.44)	10.30*** 1.28 [0.95, 1.60]
Directive language	1.58 (0.42)	1.78 (0.55)	− 2.62** − 0.32 [− 0.57, − 0.07]
Collaborative language	2.37 (0.45)	1.42 (0.46)	12.57*** 1.56 [1.19, 1.92]

Degrees of freedom (dfs) = 64. ***p < 0.001, **p < 0.01

The principal components analyses (PCAs) yielded a four-component solution for chatbot responses and a three-component solution for physician responses (Appendix B: eTable 4).

For actual chatbot responses, the first component retained was *relationship-oriented,* which included the following codes: more validation, reassurance, personalized active listening, praising patient for seeking help, incorporation of psychosocial/emotional information, and non-judgmental language. The second component was *conscientious,* which included appearing not hurried or rushed and using more collaborative language. The third component was *guiding,* which included using more structured responses (i.e., presence of step-by-step guidance and a clear breakdown of actions to take) and directive language. The final component was *technical,* which included more biomedical information and medical jargon.

For actual physician responses, the first component retained was also *relationship-oriented,* which included all codes in the chatbot relationship-oriented component as well as not appearing hurried or rushed. The second component for actual physician responses was *technical*, which overlapped completely with the chatbot *technical* component. The third and final component for physicians was *structured,* which included encourages follow-up and more structured responses.

In a multiple regression predicting the online participants’ empathy ratings of chatbot responses, these four components explained 22% of the variance in chatbot empathy ratings, with *relationship-oriented* and *conscientious* being significant positive predictors of empathy (Appendix B: eTable 5). The three components retained for actual physician responses explained 62% of the variance in actual physician empathy ratings, with *relationship-oriented* and *structured* being significant positive predictors of empathy (Appendix B: eTable 5).

## DISCUSSION

Using a large sample of online participants, we replicated Ayers et al.’s findings for responses to patient questions in a public online forum such that chatbot responses were rated as more empathic and of higher quality than physician responses. We extended this work by examining ratings of liking, trust, and how good the response was, all of which were rated higher when the response was authored by a chatbot compared to a physician. Importantly, having information about who authored the response mattered, as participants rated responses they believed to be from a physician higher in empathy than responses they believed to be generated by a chatbot, regardless of the actual source of the response. Participants perceived the highest levels of empathy when they were told a physician had authored the response, but it was actually generated by a chatbot.

We also compared actual chatbot responses to actual physician responses by coding specific elements of verbal behavior that could have been present within the online written responses. Compared to physician responses, chatbot responses included more validation, active listening, encouragement of follow-up, structured responses, non-judgmental language, praise for the patient’s help seeking, psychosocial/emotional and biomedical information, and collaborative language, and they included less directive language and appeared less hurried than physician responses.

Our findings are consistent with past research demonstrating that chatbot responses were better at detecting emotions and offering emotional support than human-generated responses.^24^ In that study, recipients of chatbot responses felt more heard than recipients of human-generated responses when they did not know who authored the response. However, consistent with our findings, when participants learned a chatbot had authored the response, they felt less heard than when they learned a human had authored the response.^24^ Our work extends this by directly comparing chatbot and physician responses using real-world patient questions and identifying specific verbal features linked to perceived empathy.

While empathy is only one dimension to consider in both physician responses and AI-generated responses, our study showed that ratings of trust, liking, goodness, and quality mirrored ratings of empathy; however, other dimensions deserve examination. For example, in one small-scale study of responses to patient questions on an “Ask the Rheumatologist” site, rheumatology patients (*n* = 17) rated chatbot responses to questions similarly to physician-generated responses in terms of comprehensiveness, readability, and overall preference while rheumatologists (*n* = 4) rated chatbot responses *lower* than physician responses on all three dimensions.^25^

### Limitations and Future Research

The current work suggests that chatbots may be a tool for physicians to augment responses to patients, for example through secure virtual messaging portals. AI-generated messages may be perceived as empathic, trustworthy, and high quality. However, it is vital to keep in mind that the physician responses used in the present study likely are not a fair representation of how physicians in their actual medical practice—that is, in a professionally sanctioned (therefore responsible and accountable) relationship to a patient—would behave. The present physician responses were given anonymously to strangers in an online forum, under uncontrolled circumstances and in voluntary, perhaps haphazard, circumstances. This context likely does not reflect how physicians communicate with their own patients in clinical settings, where tone, detail, and relational depth may differ significantly. We chose to use the physician and chatbot responses from a previously published study^4^ to support replication and extension of empathy ratings; however, we encourage additional studies which examine AI-generated responses in clinical notes or messaging available in patient portals and other technology-mediated communication. Importantly, the value of the present work is therefore not in showing that the real physicians were inadequate, but rather in demonstrating that AI-generated responses can be perceived as empathic along multiple dimensions.

In addition to this online reddit forum not representing all communication between physicians and patients, our participants’ ratings may not be generalizable across different patient populations. Using online participants to make ratings of communication is a widely accepted, validated methodology in clinical communication research.^26,27^ However, future research should explore how different patient populations might differ in their reactions to AI-generated communication. Patient demographic and clinical characteristics, including age, race, and disease, have all been shown to influence willingness to embrace AI tools for clinical diagnosis.^28^ Future research should also examine how an existing relationship with a physician might change the way responses augmented with AI are viewed by patients. It may be that participants in this study rated actual chatbot responses as higher in empathy because the phrases and elements in these responses felt unexpected or uncommon, relative to their usual communication with physicians. As AI enables or auto-populates these elements of empathy into more technology-mediated written clinical communication, or as physicians learn how to convey empathy more effectively themselves, the physician-AI gap in rated empathy may grow smaller. Similarly, if a physician is not using these empathic elements during in-person communication with the patient, and then they appear in technology-mediated communication, the patient may not view the AI-generated communication or their physician as positively.

We also caution against using these results to support blindly adopting AI-generated patient communication. Deliberate human oversight is crucial as public AI tools such as ChatGPT have been known to produce AI hallucinations, fabricating non-existent patient information or generating incorrect or misleading information. It is also likely that biases found in human-generated text (e.g., race and gender biases) may be transferred and augmented in AI-generated responses resulting in biased responses and decision-making. Additionally, we feel it is an ethical imperative to disclose to patients that chatbots assisted in the generation of responses with human oversight, even though our findings demonstrate that such transparency has the potential to reduce the amount of empathy and other good qualities perceived by patients.^29^ Much more work is needed to understand how implementing human-directed chatbot responses into clinical practice impacts a myriad of outcomes including patient satisfaction, health outcomes, and physician burnout and job satisfaction. Additionally, future research should assess the correctness and clinical accuracy of chatbot vs. physician responses to patient questions, as this was not the focus of the current work.

Clinical training around expressing empathy can be enhanced from this work. For example, medical students and trainees can learn to incorporate specific elements that were generated from chatbots and rated as more empathic, including: validating emotions (“It’s understandable that you’re feeling anxious”), providing reassurance (“It’s a good sign that your tests came back normal”), displaying active listening (“You mentioned that this has been going on for several months…”), or offering praise (“You’ve done the right thing by seeking care”). For physicians, AI-augmented messaging that includes such phrases may help physicians learn to incorporate them in their communication over time without needing to use AI. These additions to communication can convey empathy, which improves the patient experience and patient outcomes.^7^

## CONCLUSIONS

It remains an open, and somewhat controversial, question whether AI can replace the human connection between physicians and their patients.^30^ However, AI-generated responses include specific verbal elements that are perceived as empathic. When used ethically and with full disclosure to patients, physicians who learn to integrate AI into their communication and practice may eventually outperform those who do not. As generative AI is increasingly integrated in electronic medical record systems, patient portals, and technology-mediated clinical communication, it will be imperative to continue to examine patients’ perceptions and experiences to optimize care.

## Supplementary Information

Below is the link to the electronic supplementary material.

Supplementary Material 1 (XLSX 70.5 KB)

Supplementary Material 2 (DOCX 30.6 KB)

Supplementary Material 3 (DOCX 15.3 KB)

## Data Availability

Data and materials will be made available upon email request to the corresponding author MAR.

## References

[1] **Mehrotra A, Ray K, Brockmeyer DM, Barnett ML, Bender JA.** Rapidly Converting to “Virtual Practices”: Outpatient Care in the Era of Covid-19. Catalyst non-issue content. 2020;1(2)10.1056/CAT.20.0091

[2] **Shanafelt TD, Hasan O, Dyrbye LN, et al.** Changes in Burnout and Satisfaction With Work-Life Balance in Physicians and the General US Working Population Between 2011 and 2014. Mayo Clin Proc. 2015;90(12):1600-13. 10.1016/j.mayocp.2015.08.023 26653297 10.1016/j.mayocp.2015.08.023

[3] **Al Kuwaiti A, Nazer K, Al-Reedy A, et al.** A Review of the Role of Artificial Intelligence in Healthcare. J Pers Med. 2023;13(6)10.3390/jpm13060951 10.3390/jpm13060951 PMC10301994 37373940

[4] **Ayers JW, Poliak A, Dredze M, et al.** Comparing Physician and Artificial Intelligence Chatbot Responses to Patient Questions Posted to a Public Social Media Forum. JAMA Intern Med. 2023;183(6):589-596. 10.1001/jamainternmed.2023.1838 37115527 10.1001/jamainternmed.2023.1838 PMC10148230

[5] **Perry A.** AI Will Never Convey the Essence of Human Empathy. Nat Hum Behav. 2023;11:1808-1809. 10.1038/s41562-023-01675-w 37474839

[6] **Morse DS, Edwardsen EA, Gordon HS.** Missed Opportunities for Interval Empathy in Lung Cancer Communication. Arch Intern Med. 2008;168(17):1853-1858. 10.1001/archinte.168.17.1853 18809811 10.1001/archinte.168.17.1853 PMC2678758

[7] **Nembhard IM, David G, Ezzeddine I, Betts D, Radin J.** A Systematic Review of Research on Empathy in Health Care. Health Serv Res.2023;58(2):250-263. 10.1111/1475-6773.14016 35765156 10.1111/1475-6773.14016 PMC10012244

[8] **Hall JA, Duong F, Schwartz R.** On the Proliferation of the Empathy Concept in Healthcare and Medical Education esearch. Patient Educ Couns. 2024;119:108041. 10.1016/j.pec.2023.108041 37945425 10.1016/j.pec.2023.108041

[9] **Hall JA, Schwartz R.** Empathy Present and Future. J Soc Psychol. 2019;159(3):225-243. 10.1080/00224545.2018.1477442 29781776 10.1080/00224545.2018.1477442

[10] **Hall JA, Schwartz R.** Empathy, an Important but Problematic Concept. J Soc Psychol. 2022;162(1):1-6. 10.1080/00224545.2021.2004670 34978951 10.1080/00224545.2021.2004670

[11] **Hall JA, Schwartz R, Duong F, et al.** What is Clinical Empathy? Perspectives of Community Members, University Students, Cancer Patients, and Physicians. Patient Educ Couns. 2021;104(5):1237-1245. 10.1016/j.pec.2020.11.001 33234440 10.1016/j.pec.2020.11.001

[12] **Gerger H, Munder T, Kreuzer N, Locher C, Blease C.** Lay Perspectives on Empathy in Patient-Physician Communication: An Online Experimental Study. Health Commun. 2024;39(6):1246-1255. 10.1080/10410236.2023.2210380 37219394 10.1080/10410236.2023.2210380

[13] **Sanders JJ, Dubey M, Hall JA, Catzen HZ, Blanch-Hartigan D, Schwartz R.** What is Empathy? Oncology Patient Perspectives on Empathic Clinician Behaviors. Cancer. 2021;127(22):4258-4265. 10.1002/cncr.33834 34351620 10.1002/cncr.33834

[14] **Cuff B, Brown SJ, Taylor L, Howat DJ.** Empathy: A Review of the Concept. Emot Rev. 2016;8(2):144-153. 10.1177/1754073914558466

[15] **Sulzer SH, Feinstein NW, Wendland CL.** Assessing Empathy Development in Medical Education: A Systematic Review. Med Educ. 2016;50(3):300-10. 10.1111/medu.12806 26896015 10.1111/medu.12806 PMC4914035

[16] **Mercer SW, Reynolds WJ.** Empathy and Quality of Care. Br J Gen Pract. 2002;52 Suppl(Suppl):S9–12. PMC1316134 12389763

[17] **Hojat M, DeSantis J, Shannon SC, et al.** The Jefferson Scale of Empathy: a nationwide study of measurement properties, underlying components, latent variable structure, and national norms in medical students. Adv Health Sci Educ Theory Pract. 2018;23(5):899-920. 10.1007/s10459-018-9839-9 29968006 10.1007/s10459-018-9839-9 PMC6245107

[18] **Morse JM, Anderson G, Bottorff JL, et al.** Exploring empathy: a conceptual fit for nursing practice? Image J Nurs Sch. Winter 1992;24(4):273-80. 10.1111/j.1547-5069.1992.tb00733.x 10.1111/j.1547-5069.1992.tb00733.x 1452181

[19] **Westendorp J, Stouthard J, Meijers MC, et al.** The power of clinician-expressed empathy to increase information recall in advanced breast cancer care: an observational study in clinical care, exploring the mediating role of anxiety. Patient Educ Couns. 2021;104(5):1109-1115. 10.1016/j.pec.2020.10.025 33168460 10.1016/j.pec.2020.10.025

[20] **Links M, Ayling T, Doran J, et al.** A compassionate pause. Patient Educ Couns. 2021;104(2):432-436. 10.1016/j.pec.2020.08.012 32873444 10.1016/j.pec.2020.08.012

[21] **Douglas BD, Ewell PJ, Brauer M.** Data quality in online human-subjects research: Comparisons between MTurk, Prolific, CloudResearch, Qualtrics, and SONA. PLoS One. 2023;18(3):e0279720. 10.1371/journal.pone.0279720 36917576 10.1371/journal.pone.0279720 PMC10013894

[22] **Abbey JD, Meloy MG.** Attention by design: Using attention checks to detect inattentive respondents and improve data quality. J Oper Manag. 2017;53–56:63–70. 10.1016/j.jom.2017.06.001

[23] **Park HS, Dailey R, Lemus D.** The use of exploratory factor analysis and principal components analysis in communication research. Human communication research. 2002;28(4):562-577.

[24] **Yin Y, Jia N, Wakslak CJ.** AI can help people feel heard, but an AI label diminishes this impact. Proc Natl Acad Sci U S A. 2024;121(14):e2319112121. 10.1073/pnas.2319112121 38551835 10.1073/pnas.2319112121 PMC10998586

[25] **Ye C, Zweck E, Ma Z, Smith J, Katz S.** Doctor Versus Artificial Intelligence: Patient and Physician Evaluation of Large Language Model Responses to Rheumatology Patient Questions in a Cross-Sectional Study. Arthritis Rheumatol. 2024;76(3):479-484. 10.1002/art.42737 37902018 10.1002/art.42737

[26] **Blanch-Hartigan D, Hall JA, Krupat E, Irish JT.** Can naive viewers put themselves in the patients' shoes?: reliability and validity of the analogue patient methodology. Med Care. 2013;51(3):e16-21. 10.1097/MLR.0b013e31822945cc 22498688 10.1097/MLR.0b013e31822945cc

[27] **van Vliet LM, van der Wall E, Albada A, Spreeuwenberg PM, Verheul W, Bensing JM.** The validity of using analogue patients in practitioner-patient communication research: systematic review and meta-analysis. J Gen Intern Med. 2012;27(11):1528-43. 10.1007/s11606-012-2111-8 22700392 10.1007/s11606-012-2111-8 PMC3475831

[28] **Robertson C, Woods A, Bergstrand K, Findley J, Balser C, Slepian MJ.** Diverse patients' attitudes towards Artificial Intelligence (AI) in diagnosis. PLOS Digit Health. 2023;2(5):e0000237. 10.1371/journal.pdig.0000237 37205713 10.1371/journal.pdig.0000237 PMC10198520

[29] **Haltaufderheide J, Ranisch R.** The ethics of ChatGPT in medicine and healthcare: a systematic review on Large Language Models (LLMs). NPJ Digit Med. 2024;7(1):183. 10.1038/s41746-024-01157-x 38977771 10.1038/s41746-024-01157-x PMC11231310

[30] **Inzlicht M, Cameron CD, D'Cruz J, Bloom P.** In praise of empathic AI. Trends Cogn Sci. 2024;28(2):89-91. 10.1016/j.tics.2023.12.003 38160068 10.1016/j.tics.2023.12.003

