# Supplementary material for: What is Artificial Intelligence (AI) “Empathy”? A Study Comparing ChatGPT and Physician Responses on an Online Forum
Source: J Gen Intern Med. 2025 Dec 8;41(5):1304–11. doi: 10.1007/s11606-025-10068-w (PMC13019702; doi:10.1007/s11606-025-10068-w)
Supplement: Supplementary file 2 — Supplementary Material 2 (DOCX 30.6 KB) [file 11606_2025_10068_MOESM2_ESM.docx]

**Supplemental Appendix B**

eTable 1. Definitions of Physician and Chatbot Response Codes

| **Code** | **Definition** |
| --- | --- |
| **Validation of emotion/experience or expressing concern**  (chatbot α = 0.95)  (physician α = 0.97) | Acknowledging patient emotion or experience or expressing concern (e.g., “I can imagine this must be concerning for you” or “It’s understandable that you’re feeling anxious” or “That must be hard for you…” or “It’s really difficult when…”) |
| **Reassurance**  (chatbot α =0 .88)  (physician α = 0.74) | Statements aimed at reducing worry (e.g., “It’s a good sign that your tests came back normal” or “This sounds manageable”) |
| **Personalized/active listening**  (chatbot α = 0.89)  (physician α = 0.88) | Tailoring responses to specific patient details, reflecting personal understanding, reflecting back what the patient said (e.g., “Since you’ve mentioned you’ve been through a tough time with your dental care…” or “Given your concerns about your child’s health…” or “You mentioned that this has been going on for several months…”) |
| **Encourages follow-up**  (chatbot α = 0.96)  (physician α = 0.93) | Encouraging the patient to ask further questions or seek medical care (e.g., “I recommend checking in with your healthcare provider for more clarity”) |
| **Structured responses**  (chatbot α = 0.78)  (physician α = 0.89) | Presence of step-by-step guidance and a clear breakdown of actions to take (e.g., “First, you can do X. Then, you should consider Y…”) |
| **Non-judgmental language**  (chatbot α = 0.73)  (physician α = 0.73) | Avoidance of harsh or dismissive language. Instead, using non-judgmental terms (e.g., “It’s great that you’re thinking about seeing a dentist” vs. “You should have gone to a dentist sooner”) |
| **Praising patient for seeking help**  (chatbot α = 0.70)  (physician α = 0.71) | Praising the patient for taking action or seeking help (e.g., “It’s great that you reached out about this” or “You’ve done the right thing by seeking care”) |
| **Use of medical jargon**  (chatbot α = 0.81)  (physician α = 0.82) | Used specialized terminology, language, or phrases that are technical, complex, or difficult for individuals without medical training to understand (e.g., "hypertension" instead of "high blood pressure" or "myocardial infarction" instead of "heart attack") |
| **Gave off impression of being hurried or rushed**  (chatbot α = 0.96)  (physician α = 0.98) | Response delivered quickly, without much attention to detail or thoroughness |
| **Incorporation of psychosocial/emotional information**  (chatbot α = 0.88)  (physician α = 0.90) | Addresses aspects of the patient’s life beyond the physical or medical condition such as relationships, work, finances, culture, emotions, stress, and emotional wellbeing, among others |
| **Incorporation of biomedical information**  (chatbot α = 0.79)  (physician α = 0.78) | Focus on the physical and biological aspects of the patient's condition such as clinical data, medical procedures, or physiological explanations |
| **Directive language**  (chatbot α = 0.68)  (physician α = 0.60) | Gives clear, specific instructions on what the patient should do (e.g., “You need to get an ultrasound”) |
| **Collaborative language**  (chatbot α = 0.87)  (physician α = 0.87) | Engages the patient in decision-making by giving options that emphasize shared decision-making and mutual respect (e.g., “It might be helpful to consider an ultrasound”) |
| *Note*. α is interrater reliability across coders. | |

eTable 2. Intercorrelations of Participant Ratings by Response Condition.

| **Told Chatbot Actual Chatbot** | | | | | |
| --- | --- | --- | --- | --- | --- |
|  |  | **Quality** | **Empathy** | **Liking** | **Trust** |
|  | **Quality** |  |  |  |  |
|  | **Empathy** | 0.48*** |  |  |  |
|  | **Liking** | 0.73*** | 0.62*** |  |  |
|  | **Trust** | 0.72*** | 0.58*** | 0.64*** |  |
|  | **Good** | 0.90*** | 0.58*** | 0.79*** | 0.80*** |
| **Told Chatbot Actual Physician** | | | | | |
|  | **Quality** |  |  |  |  |
|  | **Empathy** | 0.77*** |  |  |  |
|  | **Liking** | 0.70*** | 0.76*** |  |  |
|  | **Trust** | 0.86*** | 0.81*** | 0.77*** |  |
|  | **Good** | 0.91*** | 0.79*** | 0.78*** | 0.89*** |
| **Told Physician Actual Physician** | | | | | |
|  | **Quality** |  |  |  |  |
|  | **Empathy** | 0.72*** |  |  |  |
|  | **Liking** | 0.82*** | 0.77*** |  |  |
|  | **Trust** | 0.79*** | 0.62*** | 0.76*** |  |
|  | **Good** | 0.74*** | 0.64*** | 0.71*** | 0.72*** |
| **Told Physician Actual Chatbot** | | | | | |
|  | **Quality** |  |  |  |  |
|  | **Empathy** | 0.49*** |  |  |  |
|  | **Liking** | 0.71*** | 0.71*** |  |  |
|  | **Trust** | 0.72*** | 0.57*** | 0.70*** |  |
|  | **Good** | 0.78*** | 0.64*** | 0.82*** | 0.77*** |
| *Note.* ****p* < .001. | | | | | |

eTable 3. Comparison of Ayers et al. and Subsample of 65 Responses Used in Present Study on Ratings of Quality by Actual Chatbots and Actual Physicians.

|  | **Quality of Actual Chatbot Response**  ***M* (*SD*)** | **Quality of Actual Physician Response**  ***M* (*SD*)** | **Paired samples *t*-test**  ***t*(df), *p*-value**  **Cohen’s *d*** |
| --- | --- | --- | --- |
| Ayers et al. physician ratings | 4.12 (0.51) | 3.34 (0.66) | *t*(64) = 8.58, *p* < 0.001  Cohen’s *d* = 1.07 |
| Current study participant ratings | 3.87 (0.66) | 3.22 (0.64) | *t*(64) = 5.09, *p* < 0.001  Cohen’s *d* = 0.64 |

eTable 4. Components and Factor Loadings of Principal Components Analysis for Chatbot Empathy Response Codes and Physician Empathy Response Codes.

|  | Components | | | | |
| --- | --- | --- | --- | --- | --- |
| Code | **Relationship-Oriented**  **(α_c_ = 0.90)**  **(α_p_ = 0.90)** | **Conscientious**  **(α_c_ = 0.63)** | **Guiding**  **(α_c_ = 0.32)** | **Technical**  **(α_c_ = 0.65)**  **(α_p_ = 0.61)** | **Structured**  **(α_p_ = 0.47)** |
| Validation of emotion/experience | C = 0.89  P = 0.90 |  |  |  |  |
| Reassurance | C = 0.76  P = 0.81 |  |  |  |  |
| Personalized/active listening | C = 0.84  P = 0.68 |  |  |  |  |
| Praising patient for seeking help | C = 0.81  P = 0.83 |  |  |  |  |
| Incorporation of psychosocial/emotional information | C = 0.85  P = 0.82 |  |  |  |  |
| Non-judgmental language | C = 0.65  P = 0.73 |  |  |  |  |
| Encourages follow-up |  |  |  |  | P = 0.78 |
| Not hurried or rushed | P = 0.67 | C = 0.70 |  |  |  |
| Collaborative language |  | C = 0.64 |  |  |  |
| Structured responses |  |  | C = 0.66 |  | P = 0.79 |
| Directive language |  |  | C = 0.80 |  |  |
| Incorporation of biomedical information |  |  |  | C = 0.82  P = 0.87 |  |
| Medical jargon |  |  |  | C = 0.81  P = 0.79 |  |
| *Note*. Degrees of freedom (dfs) = 64. ****p* < 0.001, ***p* < 0.01. C represents factor loadings for a chatbot empathy response component while P represents factor loadings for a physician empathy response component. | | | | | |

eTable 5. Unstandardized and Standardized Betas in Multiple Linear Regressions Examining Effects of Coded Components on Chatbot Empathy Ratings and Physician Empathy Ratings.

| **Predictor Variable** | **Chatbot Empathy Ratings** | | | | **Physician Empathy Ratings** | | | |
| --- | --- | --- | --- | --- | --- | --- | --- | --- |
|  | *B*  (β) | *t* | Lower 95% CI | Upper 95% CI | *B*  (β) | *t* | Lower 95% CI | Upper 95% CI |
| **Relationship-Oriented** | 0.52  (0.29) | 2.15* | 0.04 | 1.00 | 1.37  (0.65) | 7.68*** | 1.01 | 1.72 |
| **Conscientious** | 0.75  (0.28) | 2.19* | 0.07 | 1.44 | --- | --- | --- | --- |
| **Guiding** | 0.42  (0.19) | 1.62 | -0.10 | 0.93 | --- | --- | --- | --- |
| **Technical** | -0.13  (-0.06) | -0.49 | -0.67 | 0.41 | 0.15  (0.07) | 0.89 | -0.19 | 0.49 |
| **Structured** | --- | --- | --- | --- | 0.44  (0.25) | 2.96** | 0.14 | 0.74 |
| R-Squared | 0.27 |  |  |  | 0.64 |  |  |  |
| Adjusted R-Squared | 0.22 |  |  |  | 0.62 |  |  |  |
| *F* | 5.37*** |  |  |  | 35.38*** |  |  |  |
| *Note*. Chatbot Empathy Ratings were empathy ratings when participants perceived a chatbot response and it was an actual chatbot response, Physician Empathy Ratings were empathy ratings when participants perceived a physician response and it was an actual physician response. Structured was not a component for Chatbot Empathy Ratings and Conscientious and Guiding were not components for Physician Empathy Ratings. *B* is unstandardized beta coefficient and β is standardized beta coefficient. ****p* < 0.001, ***p* < 0.01, **p* < 0.05. | | | | | | | | |
