# Supplementary material for: What is Artificial Intelligence (AI) “Empathy”? A Study Comparing ChatGPT and Physician Responses on an Online Forum
Source: J Gen Intern Med. 2025 Dec 8;41(5):1304–11. doi: 10.1007/s11606-025-10068-w (PMC13019702; doi:10.1007/s11606-025-10068-w)
Supplement: Supplementary file 3 — Supplementary Material 3 (DOCX 15.3 KB) [file 11606_2025_10068_MOESM3_ESM.docx]

INSTRUCTIONS: For this activity, you will read 1 question posed by a real patient in an online forum on reddit called AskDocs where people can post their medical questions. 

We want you to imagine you are the patient asking the question.

| **The quality of information provided in the response?** | | | | |
| --- | --- | --- | --- | --- |
| Very poor | Poor | Acceptable | Good | Very good |
| 1 | 2 | 3 | 4 | 5 |
|  | | | | |
| **The empathy or bedside manner provided in the response?** | | | | |
| Not empathetic | Slightly empathetic | Moderately empathetic | Empathetic | Very empathetic |
| 1 | 2 | 3 | 4 | 5 |
|  | | | | |
| **How much would you personally like to have a doctor respond to you like this?** | | | | |
| Not at all like | Slightly like | Moderately like | Like | Like a lot |
| 1 | 2 | 3 | 4 | 5 |
|  | | | | |
| **How much do you trust this response?** | | | | |
| Not trust at all | Slightly trust | Moderately trust | Trust | Completely trust |
| 1 | 2 | 3 | 4 | 5 |
|  | | | | |
| **How good was this response?** | | | | |
| Not at all good | Slightly good | Moderately good | Good | Very good |
| 1 | 2 | 3 | 4 | 5 |
|  | | | | |

Please read the entire question and response and then make ratings about the response as if you were the real patient asking the question.
